# Adherence of Type 2 Diabetic Patients to Self-Care Activity: Tertiary Care Setting in Saudi Arabia

**DOI:** 10.1155/2020/4817637

**Published:** 2020-10-06

**Authors:** Ali Hassan Alhaiti, Mohammed Senitan, Wireen Leila T. Dator, Chandrakala Sankarapandian, Nadiah Abdulaziz Baghdadi, Linda Katherine JONES, Cliff Da Costa, George Binh Lenon

**Affiliations:** ^1^Nursing Department, King Fahad Medical City, Riyadh, Saudi Arabia; ^2^Department of Public Health, Faculty of Health Sciences, Saudi Electronic University, Saudi Arabia; ^3^College of Nursing, Princess Nourah Bint Abdulrahman University, Riyadh, Saudi Arabia; ^4^School of Science, RMIT University, Bundoora West Campus, Victoria 3083, Australia

## Abstract

**Aim:**

To analyse the prevalence of self-care practices in T2D patients in KSA.

**Methods:**

The study was conducted in King Fahad Medical City (KFMC) in Saudi Arabia, and 385 patients were selected as samples. Data were collected using the Summary of Diabetes Self-Care Activities-Arabic (SDSCA) and consisted of 14 items related to self-care activities of T2D patients related to management and control of disease and four other aspects related to education and advice from healthcare members regarding management of T2D.

**Results:**

The self-care attributes including adherence to medication commitment activities (*M* = 6.13, SD = 1.25) were the most practised of all the domains. Glucose monitoring (*M* = 4.15, SD = 2.42) and foot care (*M* = 3.28, SD = 1.69) were at an average level, and adherence to the diet plan and exercise was found to be at a poor level (*M* = 2.57, SD = 1.73 and *M* = 2.13, SD = 2.00) respectively. About 179 patients (74.3%) were found to be advised to follow a low-fat eating plan, and only 89 patients (36.9%) had received information concerning fruits and vegetables in their diet. More than 90% patients were found to be advised to strictly carry out exercise and blood sugar monitoring.

**Conclusion:**

It was found that adherence to self-care activities including diet, exercise, and foot care was relatively poor while intake of medication was strictly followed. The education provided by healthcare providers related to self-management attributes was found to be significant and had positive effects on the overall health and well-being of T2D patients.

## 1. Background

Self-activities help ensure that there is a reduction of the effects of T2D on the human body. According to Abu Sabbah and Al-Shehri [1], good self-care helps maintain good glycaemic control. This means that there is a need for patients to adhere to the various self-care activities that are recommended to them by their healthcare professionals. This includes observing various patient behaviours that are expected, including living a healthy lifestyle that includes physical exercise, healthy eating, reduction in tobacco consumption, and weight management. Another change that is expected is carrying out various disease self-management activities such as self-medication and self-monitoring of glucose levels.

In most cases, patients need to implement various self-care activities either for themselves or with the help of close family members. Kamel et al. [2] argue that more than 95% of T2D cases require self-care activities that include changing their lifestyles to reduce the complications that are associated with the illness. Hence, patients require a good understanding of self-care activities that they should observe on a daily basis so that they can survive the associated complications of the disease.

Research has found that around a third of patients do not have an understanding of the various early symptoms of the illness. According to Jordan and Jordan [3], there is a need for patients to understand the various symptoms of the disease at different stages so that they know which self-care activities they need to carry out at any given time. According to research, there is poor adherence to various self-monitoring activities that T2D patients are expected to undertake. According to Mohd et al. [4], a range of factors affect the level of adherence to self-care activities and these may be social, economic, or cultural.

Family support is very important in ensuring that there is adherence to self-care activities among patients. Al-Aboudi et al. [5] argue that families provide motivation that plays a therapeutic role and hence ensures that there is an increase in adherence to self-care activities. Accordingly, there is a high adherence level among patients who have good family support, compared with patients who have less family support. Family and friends support patients to ensure they are motivated to make the right decisions, follow various recommendations made by healthcare professionals, and undertake the various lifestyle changes that are expected.

One of the ways in which patients can be motivated to adhere to various self-care management activities is through the use of nursing consultants who can provide individuals with the necessary training for self-care and self-monitoring. Aljohani et al. [6] argue that nursing consultants ensure that patients are well equipped with the necessary skills and knowledge about the need to carry out various self-care activities and to change their lifestyle to ensure they suffer fewer complications from the disease.

## 2. Methodology

The study was conducted in King Fahad Medical City (KFMC) in Saudi Arabia which is considered one of the largest healthcare facilities in the Gulf Region, with a total capacity of 1,200 beds. A convenience sample of 385 participants was targeted for recruitment. However, to recruit participants, the researcher first obtained permission from the head of the Diabetes Centre (SDEC). As part of patient recruitment procedures, the researcher remained in the Diabetes Centre of KFMC's and approached patients who were receiving educational support on their T2D diagnosis.

Data were collected using the Summary of Diabetes Self-Care Activities-Arabic (SDSCA). Permission to use the SDSCA was obtained from Aljohani et al. [7]. The instrument is composed of two parts. The first part consists of 14 items related to self-care activities to measure patient behaviour concerning the self-management of T2D with regard to five subscales: diet (three items), exercise (two items), blood glucose testing (two items), foot care (five items), and medication (two items). Each item asks about the number of days (0–7) on which the patient did a certain activity (related to diabetes care) within the last week.

The second part is the extended SDSCA survey which investigates four main aspects (domains): whether diabetic patients receive advice from the healthcare members regarding diet; whether diabetic patients receive advice from the healthcare members regarding exercise; whether diabetic patients receive advice from the healthcare members regarding the monitoring of glucose levels; and the type of medication prescribed to diabetic patients by doctors.

## 3. Result

### 3.1. Demographic Characteristics

Demographic data collected from the patients included their A1C level, age group, gender, monthly income, smoking status, time since diagnosis, education level, and presence of other diseases. As shown in Table 1, the majority of patients had been diagnosed with T2D for more than 10 years (*n* = 76, 30.8%); 29.6% had been diagnosed 8–10 years prior to the study (*n* = 73); and only 4.9% had been diagnosed less than 2 years prior (*n* = 12). Nearly half of the patients had at least one disease other than diabetes (*n* = 94, 38.1%); 22.7% had only T2D (*n* = 56). Half of the patients had poor diabetic control as demonstrated by the A1C test (*n* = 122, 49.4%).

### 3.2. Patient Adherence to the Type 2 SDSCA

A mean score was calculated for each subscale. A higher score indicates a higher adherence to diabetes self-care activities. As shown in Table 2, patient adherence to medication commitment activities (*M* = 6.13, SD = 1.25) was in an active status and was the most practised of all the domains. Glucose monitoring (*M* = 4.15, SD = 2.42) and foot care (*M* = 3.28, SD = 1.69) were at an average level compared with the patient behaviour in relation to diet (*M* = 2.57, SD = 1.73) and exercise (*M* = 2.13, SD = 2.00), which were at a poor level.

Overall, results show that adherence to self-care activities was relatively poor: mean adherence scores were 2.14 for exercise, 2.57 for diet, and 3.28 for foot care. The only subscale that achieved a high mean value was the medication domain (Table 2).

### 3.3. The Extended SDSCA Survey

### 3.4. Diet Domain

Results shown in Table 3 revealed that 179 patients (74.3%) had been advised to follow a low-fat eating plan, and only 89 patients (36.9%) had received information concerning fruits and vegetables in their diet.

Table 3 shows that the most frequent advice given to patients was to follow a low-fat diet (*n* = 179) while the least frequent advice was to eat lots of fruit and vegetables (*n* = 89). This may be due to high fat consumption in Saudi T2D and in T2D patients generally, which makes this advice the most common among healthcare practitioners.

### 3.5. Exercise Domain

Table 4 provides the patients' responses concerning the exercise recommendations they received from the healthcare team at the Diabetes Centre. The majority (*n* = 161, 66.8%) of patients agreed that the healthcare team had advised them to do low-level exercise such as walking on a daily basis to improve their blood sugar level. Moreover, 103 patients (42.7%) had been advised to undertake continuous exercise for at least 20 minutes at least three times a week. Finally, 219 patients (90.9%) confirmed that they had been given advice about exercise by the healthcare team.

Table 4 shows that the most commonly recommended activity by healthcare practitioners was doing low-level exercise (*n* = 161) while the least mentioned one was fitting the exercise into the daily routine (*n* = 90).

### 3.6. Blood Sugar Domain

As shown in Table 5, the majority of patients (*n* = 193, 80.1%) had been told by the healthcare team to test their blood sugar using a machine to read the result. However, only 56 patients (23.2%) had been advised to test their urine for sugar. Overall, 231 patients (95.9%) agreed they had been given advice either about testing their blood or urine sugar level by the healthcare team on various occasions.

### 3.7. Prescribed Medication Domain

This domain investigates the medication given to patients by their doctors. As shown in Table 6, 7, 8 and 9 only 73 patients (30.3%) were given an insulin shot one or two times a day, while 105 patients (43.6%) were given insulin three or more times a day and 69 (28.6%) were given pills to control their blood sugar level. Finally, 11 patients (4.6%) mentioned that they had been prescribed neither insulin nor pills for their diabetes.

### 3.8. Effect of Different Confounders on Type 2 Diabetes Medication

According to the regression model summary, *R*‐squared = 0.053, implying that 5.3% of the regression of type 2 diabetes medication on monthly income, blood sugar test, diet, educational level, exercise, age, gender, and foot care was explained by the model. This depicted a weak form of explanation between the predictors and the response variable.

On the regression analysis of variance, there was evidence for model inadequacy associated with type 2 medication (*F*(8,232) = 1.622, *p* = 0.119). The analysis implied that the model proved inapplicable in explaining the effect of the monthly income, blood sugar test, diet, educational level, exercise, age, gender, and foot care on type 2 diabetes medication across the patients.

On the parametric tests, the intercept presented a positive effect on the type 2 diabetes medication (*β* = 5.475). Furthermore, the parameter proved statistically significant at 5% level of significance, hence appropriate for modelling (*t* = 10.895, *p* = 0.000). Diet of the persons had a negative effect on the type 2 diabetes medication (*β* = −0.052). The parameter proved statistically insignificant at 5% level of significance (*t* = −1.044, *p* = 0.297). Exercise presented a positive effect on the type 2 diabetes medication (*β* = 0.085). Moreover, the parameter proved statistically significant at 5% level of significance (*t* = 2.066, *p* = 0.040). Blood sugar tests had a positive effect on the type 2 diabetes medication (*β* = 0.049). The parameter proved statistically insignificant at 5% level of significance, hence not required for prediction of the type 2 diabetes medication. Foot care of the patients had a negative effect on the type 2 diabetes medication (*β* = −0.011). The parameter proved statistically insignificant at 5% level of significance (*t* = −0.219, *p* = 0.827). The medical and nutritional factors implied that diet and foot care had a negative effect on the type 2 diabetes medication as the exercise and blood sugar tests have a positive effect on the type 2 diabetes medication.

The age of an individual had a positive effect on the type 2 diabetes medication (*β* = 0.066). However, the parameter had an insignificant impact at 5% level of significance (*t* = 0.839, *p* = 0.402). Gender of the individuals presented a negative impact on the type 2 diabetes medication (*β* = −0.084). The parameter proved statistically insignificant (*t* = −0.505, *p* = 0.614). Education levels of the patients had a positive impact on the type 2 diabetes medication (*β* = 0.125). The parameter proved significant at 10% level of significance (*t* = 1.748, *p* = 0.082). Lastly, monthly income of the patients had a negative effect on the type 2 diabetes medication (*β* = −0.036). The parameter proved statistically insignificant at 5% level of significance (*t* = −0.466, *p* = 0.642). According to the demographic information, age and educational levels painted a positive effect on type 2 diabetes medication. However, gender and monthly income presented a negative impact on type 2 diabetes medication.

## 4. Discussion

This finding is similar to that in a study by Sabbah and AlShehri [1], who found that the level of compliance to diet, exercise, and foot care was poor compared with compliance to medication, which was 94.7%. The results are also similar to those reported by Sweileh et al. [8] for Northern Palestine where the rate of noncompliance among T2D patients was 6.5% (52.4% had partial compliance and 42% had good compliance). Li et al. [9] also found that foot care among T2D patients was poor and that there was a need for patients to attend periodic inspections. Doctors should also provide more information to their patients regarding foot care activities to promote positive behaviour change.

With regard to compliance with exercise, [10] reported similar results to the current study in that there was very low compliance with exercise for diabetic patients. In their study, the percentage of patients that exercised three to seven times per week was only 32.3. Studies have shown that the level of adherence to exercise among diabetic patients in the USA is only 19% [11]: in that study, only 39% of patients with T1DM and 37% of patients with T2D adhered to exercise routines. The patients who adhered poorly to medication were more likely to have poor adherence to exercise during the summer. Around half of the patients with high medication adherence, 39.8% of those who moderately adhere to medication, and 88.2% of patients with low medication adherence found it difficult to exercise in summer when the weather was hot Mohd et al. [4].

In contrast, Delamater [11] found that in one study that used a national sample of T2D patients, 80% of patients being treated by exercise and diet, 65% on oral medication, and 24% being treated with insulin did not monitor their blood glucose level daily. In yet another cross-national study conducted in the UK on T2D patients, patient adherence to SMBG was 70% among T2D patients and 64% among T1D patients [3]. Many studies have shown that only 15–70% of T2D patients engage in the recommended exercise, which requires exercising continuously for at least 20 minutes on a minimum of three occasions per week.

Although adherence to medication is relatively good in general, various factors determine the rate of medication adherence for diabetic patients. According to Mohd et al. [4], some predictors of medication adherence among diabetic patients in the UAE were insulin use, age and ethnicity, the presence of a chronic condition, gender, and academic attainment. Similarly, sociodemographic features that influenced strong adherence among Kuwaitis included being female (51.6%). Adherence among non-Kuwaitis was higher (57.2%) than for the Kuwait nationality [12].

In Kuwait, the nonadherence rate is surprisingly low, with only 26.1% of T2D patients not adhering to medication and making lifestyle changes [12]. Also, Mohd et al. [4] found that the mortality rate due to diabetes is higher among nonadherent than adherent T2D patients. However, Khan et al. [13] found that among patients in the Al Hasa Region of KSA, the adherence rate was low; hence, the researchers recommended improvements in the healthcare system, education, and health training for people with diabetes.

Of 533 respondents in one study in KSA, 51.6% had poor glycaemic control [14]. In the same survey, poorer glycaemic control was evident among patients who had T2D for more than 10 years (56.3%) than among patients who had the disease for less than 10 years (46.5%). In two separate US datasets (one from a national health survey and the other from a nutrition examination survey), 42% and 50% of T2D patients, respectively, had poor glycaemic control [15]. In the United Kingdom, two retrospective studies, in 1998 and 2002, showed that 79% and 76% of T2D patients, respectively, did not have adequate glycaemic control [14].

It is possible that the patient adherence to medication is not determined by the advice given to them by their doctors. There may be variables other than the effectiveness of communication that makes patients either more willing to adhere to self-management practices or to fail to adhere to self-management practices. For instance, other predictors of high adherence to self-management in the Hulu Langat District include the behaviour of not following the guidance of doctors during the Ramadan period and patients being weight conscious [16]. Nonadherence was high among patients who followed a traditional Arabic dress code [15] in Saudi Arabia.

From regression analysis, medical and nutritional factors such as diet and foot care had a negative impact on type 2 diabetes medication as the exercise and blood sugar tests have a positive effect on the type 2 diabetes medication [10]. The implication of this analysis showed that an improvement in diet and foot care led to a decline in type 2 diabetes medication. Furthermore, exercise and blood sugar levels' improvement resulted in better outcomes in terms of medication regarding type 2 diabetes. On the demographic factors, enhancement in age and education levels led to improvement in the medication of type 2 diabetes. However, changes in gender and monthly income led to a decline in medication associated with type 2 diabetes.

## 5. Conclusion

The self-management and care of T2D in patients is considered a vital aspect in the management of disease. The adherence of T2D patients to self-care attributes as described in type 2 SDSCA presents a clear idea about the health and physiological conditions of diabetics. So, proper education and strict adherence to SDSCA is necessary for proper management of diabetes. The adherence to 14 items (diet, exercise, blood glucose testing, foot care, and medication) related to self-care activities to measure patient behaviour concerning the self-management of T2D showed that adherence to self-care activities was relatively poor. This trend needs to be changed and proper education should be provided to T2D patients to practise self-care.

The results showed that age and education levels had a positive impact on type 2 diabetes treatment as gender and monthly income affected it negatively. Diet and foot care had a negative impact on type 2 diabetes medication as the exercise and blood sugar tests have a positive effect on the type 2 diabetes medication. The outcome shows that most of the confounders especially the demographic variables painted both negative and positive impact on T2D treatment among the patients.

The extended SDSCA survey which investigates vital aspects of self-care education of T2D patients showed that most patients were advised to do regular exercise and foot care and take care of diet and medication intake, but the actual practice was lower. It is important for patients to understand the various symptoms of the disease at different stages so that they know which self-care activities they need to carry out at any given time. Various factors affect the level of adherence to self-care activities, and these may be social, economic, or cultural. For instance, the people of KSA are exposed to high calorie and fat diet and are exposed to high temperatures; both these factors are considered a major obstacle in the self-management of T2D. High temperature discourages T2D patients from doing outdoor exercise, and diet control also becomes a challenge. This can be improved by spreading knowledge about the importance of practicing self-care and consequences of not following them. Together, healthcare providers and T2D patients can lead to better management of T2D and improve the quality of life with the least complications and physiological damages attributed to T2D. Finally, patients can be motivated to adhere to various self-care management activities through the use of nursing consultants who can provide individuals with the necessary training for self-care and self-monitoring according to the lifestyle and requirement of every patient.

## Figures and Tables

**Table 1:** Patients' demographic characteristics (*n* = 247).

Characteristics		*n*	%
Age group (years)	18–30	106	42.9
	31–45	67	27.1
	46–55	31	12.6
	>55	43	17.4

Gender	Male	124	50.2
	Female	123	49.8

Monthly income (SAR)	<5.000	109	44.1
	5.000–<10.000	82	33.2
	10.000–15.000	25	10.1
	>15.000	31	12.6

Smoking status	Currently	21	8.5
	No	207	83.8
	Yes	19	7.7

Education level	Elementary school	28	11.3
	Middle school	28	11.3
	High school	82	33.2
	Bachelor degree	103	41.7
	Postgraduate degree	6	2.4

A1C level	Good control	51	20.6
	Acceptable control	74	30.0
	Poor control	122	49.4

Other diseases	Cardiac	10	4.0
	Blood pressure	24	9.7
	Kidney	6	2.4
	Eye	57	23.1
	>1	94	38.1
	None	56	22.7

Time since diagnosis (years)	<2	12	4.9
	2–4	32	13.0
	5–7	54	21.9
	8–10	73	29.6
	>10	76	30.8

**Table 2:** Patient adherence to self-management activities.

Domain	Mean	Standard deviation
Diet	2.57	1.74
Exercise	2.14	2.01
Blood sugar test	4.18	2.43
Foot care	3.28	1.69
Medication	6.14	1.25

**Table 3:** Responses to the extended SDSCA survey diet domain.

Item	Response output			
	Yes		No	
Does the healthcare team advise you to:	*n*	%	*n*	%
Follow a low-fat eating plan?	179	74.3	62	25.7
Follow a complex carbohydrate diet?	100	41.5	141	58.5
Reduce the number of calories you eat to lose weight?	103	42.7	138	57.3
Eat lots of food high in dietary fibre?	117	48.5	124	51.5
Eat lots (at least, five servings per day) of fruit and vegetables?	89	36.9	152	63.1
Eat very few sweets?	110	45.6	131	54.4
Other	18	7.5	223	92.5
I have not been given any advice about my diet by my healthcare team	25	10.4	216	89.6

**Table 4:** Responses to the extended SDSCA survey exercise domain.

Item	Response			
	Yes		No	
Does the healthcare team advise you to:	*n*	%	*n*	%
Do low-level exercise (such as walking) on a daily basis?	161	66.8	80	33.2
Exercise continuously for at least 20 minutes at least three times a week?	103	42.7	138	57.3
Fit exercise into your daily routine (e.g., take stairs instead of elevators, park a block away, and walk)?	90	37.3	151	62.7
Engage in a specific amount, type, duration, and level of exercise?	33	13.7	208	86.3
Other	10	4.1	231	95.9
I have not been given advice about exercise by healthcare team	22	9.1	219	90.9

**Table 5:** Response to the extended SDSCA survey sugar monitoring domain.

Item	Response			
	Yes		No	
Does the healthcare team advise you to:	*n*	%	*n*	%
Test your blood sugar using a machine to read the result?	193	80.1	48	19.9
Test your urine for sugar?	56	23.2	185	76.8
Other	0	0	241	100.0
I have not been given any advice either about testing my blood or urine sugar level by my healthcare team	10	4.1	231	95.9

**Table 6:** Responses to the extended SDSCA survey medication domain.

Item	Response			
	Yes		No	
Has your doctor prescribed you:	*n*	%	*n*	%
An insulin shot 1 or 2 times a day?	73	30.3	168	69.7
An insulin shot 3 or more times a day?	105	43.6	136	56.4
Diabetes pills to control your blood sugar level?	69	28.6	172	71.4
Other	12	5.0	229	95.0
I have not been prescribed either insulin or pills for my diabetes	11	4.6	230	95.4

**Table 7:** Regression summary.

Model summary				
Model	*R*	*R*-squared	Adjusted *R*-squared	Std. error of the estimate
1	0.230^a^	0.053	0.020	1.24133

^a^Predictors: constant, monthly income, blood sugar test, diet, educational level, exercise, age, gender, and foot care.

**Table 8:** Regression analysis of variance.

ANOVA^a^						
Model		Sum of squares	df	Mean square	*F*	Sig.
1	Regression	19.992	8	2.499	1.622	0.119^b^
	Residual	357.489	232	1.541		
	Total	377.481	240			

^a^Dependent variable: medication. ^b^Predictors: constant, monthly income, blood sugar test, diet, educational level, exercise, age, gender, and foot care.

**Table 9:** Regression model coefficients.

Coefficients^a^						
Model		Unstandardised coefficients		Standardised coefficients	*t*	Sig.
		*B*	Std. error	Beta		
1	Constant	5.475	0.502		10.895	0.000
	Diet	-0.052	0.050	-0.072	-1.044	0.297
	Exercise	0.085	0.041	0.136	2.066	0.040
	Blood sugar test	0.049	0.036	0.095	1.382	0.168
	Foot care	-0.011	0.052	-0.015	-0.219	0.827
	Age	0.066	0.078	0.056	0.839	0.402
	Gender	-0.084	0.167	-0.034	-0.505	0.614
	Educational level	0.125	0.072	0.114	1.748	0.082
	Monthly income	-0.036	0.078	-0.030	-0.466	0.642

^a^Dependent variable: medication.

## Data Availability

No additional data are available for sharing.
